# Supplementary material for: Retinopathy of Prematurity and Hearing Impairment in Infants Born with Very-Low-Birth-Weight: Analysis of a Korean Neonatal Network Database
Source: J Clin Med. 2021 Oct 19;10(20):4781. doi: 10.3390/jcm10204781 (PMC8537798; doi:10.3390/jcm10204781)
Supplement: Supplementary file 1 [file jcm-10-04781-s001.zip › jcm-1393260 Sup_Table_1.pdf]

Supplementary Table S1. Prevalence of retinopathy of prematurity (ROP) and visual/hearing impairment among the infants with both hearing and ophthalmic assessments.

Outcome	Prevalence
**ROP**	34.3% (982/2,863)
**Hearing impairment**	
At 18 months	2.6% (74/2,828)
At 3 years	2.0% (13/666)
**Visual impairment**	
At 18 months	0.5% (13/2,742)
At 3 years	0.5% (3/665)
