# Supplementary material for: Retinopathy of Prematurity and Hearing Impairment in Infants Born with Very-Low-Birth-Weight: Analysis of a Korean Neonatal Network Database
Source: J Clin Med. 2021 Oct 19;10(20):4781. doi: 10.3390/jcm10204781 (PMC8537798; doi:10.3390/jcm10204781)
Supplement: Supplementary file 1 [file jcm-10-04781-s001.zip › jcm-1393260 Sup_Table_2.pdf]

Supplementary Table S2. Hearing outcomes in infants treated for retinopathy of prematurity (ROP) separated based on treatment modality at follow-up visit at 18 months of age.

Hearing impairment outcomes	Laser treatment only	Anti-VEGF therapy only	Both	*P*-value*
**Overall impairment**	11 / 215 (5.1 %)	1 / 63 (1.6 %)	4 / 52 (7.7 %)	0.302
Bilateral impairment	7 (63.6%)	0	1 (25%)	0.282
**Deafness**	0	0	0	N/A

N/A=not applicable; VEGF=vascular endothelial growth factor

*Fisher's exact test or chi-square test
