# Supplementary material for: Retinopathy of Prematurity and Hearing Impairment in Infants Born with Very-Low-Birth-Weight: Analysis of a Korean Neonatal Network Database
Source: J Clin Med. 2021 Oct 19;10(20):4781. doi: 10.3390/jcm10204781 (PMC8537798; doi:10.3390/jcm10204781)
Supplement: Supplementary file 1 [file jcm-10-04781-s001.zip › jcm-1393260 Sup_Table_3.pdf]

Supplementary Table S3. Hearing impairment in children with and without visual impairment at the ages of 18 months and 3 years.

Outcomes	With visual impairment*, No./total (%)	Without visual impairment*, No./total (%)	*P*-value†
**Hearing impairment**			
At 18 months	1 / 13 (7.7)	72 / 2785 (2.6)	0.291
At 3 years	1 / 3 (33.3)	12 / 666 (1.8)	0.057

*Visual impairment at the same visit

†Fisher's exact test
